# Diffusion-weighted magnetic resonance imaging for the assessment of liver fibrosis in chronic viral hepatitis

**DOI:** 10.1371/journal.pone.0248024

**Published:** 2021-03-04

**Authors:** Phunchai Charatcharoenwitthaya, Kamonthip Sukonrut, Pornpim Korpraphong, Ananya Pongpaibul, Pairash Saiviroonporn

**Affiliations:** 1 Division of Gastroenterology, Department of Medicine, Faculty of Medicine Siriraj Hospital, Mahidol University, Bangkok, Thailand; 2 Radiology Department, Faculty of Medicine Siriraj Hospital, Mahidol University, Bangkok, Thailand; 3 Pathology Department, Faculty of Medicine Siriraj Hospital, Mahidol University, Bangkok, Thailand; Kaohsiung Medical University, TAIWAN

## Abstract

**Background:**

Accurate noninvasive methods for the assessment of liver fibrosis are urgently needed. This prospective study evaluated the diagnostic accuracy of diffusion-weighted magnetic resonance imaging (DWI) for the staging of liver fibrosis and proposed a diagnostic algorithm using DWI to identify cirrhosis in patients with chronic viral hepatitis.

**Methods:**

One hundred twenty-one treatment-naïve patients with chronic hepatitis B or C were evaluated with DWI followed by liver biopsy on the same day. Breath-hold single-shot echo-planar DWI was performed to measure the apparent diffusion coefficient (ADC) of the liver and spleen. Normalized liver ADC was calculated as the ratio of liver ADC to spleen ADC.

**Results:**

There was an inverse correlation between fibrosis stage and normalized liver ADC (p<0.05). For the prediction of fibrosis stage ≥2, stage ≥3, and cirrhosis, the area under the receiver-operating curve of normalized liver ADC was 0.603, 0.704, and 0.847, respectively. The normalized liver ADC value ≤1.02×10^−3^ mm^2^/s had 88% sensitivity, 81% specificity, 25% positive predictive value (PPV), and 99% negative predictive value (NPV) for the diagnosis of cirrhosis. Using a sequential approach with the Fibrosis-4 index followed by DWI, normalized liver ADC ≤1.02×10^−3^ mm^2^/s in patients with Fibrosis-4 >3.25 yielded an 80% PPV for cirrhosis, and a 100% NPV to exclude cirrhosis in patients with Fibrosis-4 between 1.45 and 3.25. Only 15.7% of patients would require a liver biopsy. This sequential strategy can reduce DWI examinations by 53.7%.

**Conclusion:**

Normalized liver ADC measurement on DWI is an accurate and noninvasive tool for the diagnosis of cirrhosis in patients with chronic viral hepatitis.

## Introduction

Chronic hepatitis B virus (HBV) or hepatitis C virus (HCV) infection represents a major public health problem causing substantial morbidity and mortality worldwide [1]. The prognosis and management of chronic viral hepatitis depend on the severity of liver fibrosis [2–4]. Liver biopsy has traditionally been the reference standard for determining the fibrosis stage in chronic liver disease. However, this invasive procedure carries a risk of severe complications, and its accuracy is limited by sampling heterogeneity and observer variability [5]. Several noninvasive radiological techniques have been investigated for the diagnosis of liver fibrosis and cirrhosis. It could be used to decide whether to treat a patient or defer antiviral therapy and monitor response to treatment and progression of the disease. Recently, the measurement of liver stiffness with elastographic methods has been validated to detect significant fibrosis in patients with chronic viral hepatitis [6]. Of these methods, magnetic resonance elastography has been shown to be the most accurate noninvasive method for the assessment of liver fibrosis [6]. However, it remains poorly accessible and requires special equipment. Its use is therefore currently reserved for clinical trials.

Diffusion-weighted magnetic resonance imaging (DWI) is an appealing imaging technique for the staging of liver fibrosis because it has been routinely introduced into conventional magnetic resonance imaging (MRI) protocols without the need for contrast agents. DWI is based on the intravoxel incoherent motion and has been used for the noninvasive quantification of the diffusion of water molecules in tissue, which is quantified by calculation of the apparent diffusion coefficient (ADC) [7]. Liver fibrosis, resulting from increased connective tissue in the liver, showed restriction evident on DWI. Several studies have demonstrated a significant negative correlation between ADC and the fibrosis stage [8–10]. Although ADC measurement on DWI for assessing liver fibrosis and cirrhosis has been performed, previous studies on small numbers of patients, in which various hardware and sequencing profiles were used, have reported inconsistent results for staging liver fibrosis [10–12]. Normalization of ADC using a reference organ may help reduce variability in ADC calculations. For instance, some reports showed that normalizing liver ADC with spleen ADC appeared to decrease variability of ADC and improved the diagnostic accuracy for detecting liver fibrosis and cirrhosis with better reproducibility [13, 14]. Therefore, we conducted this prospective study to evaluate the diagnostic performance of DWI with normalized liver ADC for determining the liver fibrosis stage in patients with chronic viral hepatitis with liver histology as a reference. We also propose a diagnostic algorithm using DWI to identify cirrhosis in patients with chronic viral hepatitis.

## Material and methods

### Study population

This study complied with the Declaration of Helsinki, was approved by Siriraj Institutional Review Board, and registered with ClinicalTrials.gov (NCT 02682108). The study prospectively enrolled naïve patients with chronic HBV or HCV infection who underwent a percutaneous liver biopsy in a division of gastroenterology of Siriraj Hospital. According to the reimbursement policy, liver biopsy was done as part of clinical care for staging and grading liver disease in deciding on antiviral therapy. The patients were enrolled based on the following criteria: chronic HBV infection defined as hepatitis B surface antigen positivity for more than 6 months and serum HBV DNA level greater than 2000 IU/ml; chronic HCV infection defined as the presence of anti-HCV antibodies for more than 6 months and detectable serum HCV RNA. The exclusion criteria were as follows: age <18 years; coinfection with hepatitis B and C viruses; co-existent with other liver diseases such as alcoholic, autoimmune, cholestatic, hereditary liver disease, or drug-induced liver injury; and any contraindications for MRI examination, e.g., claustrophobia, incompatible metallic implants, and pacemaker. Written informed consent was obtained from each participant before enrollment.

### Clinical and biological parameters

A comprehensive clinical assessment was performed. On the day of liver biopsy, a fasting venous blood sample was collected from all participants for aspartate aminotransferase (AST), alanine aminotransferase (ALT), complete blood count, coagulogram, glucose, total cholesterol, and triglycerides. The Fibrosis-4 (FIB-4) index was calculated using the formula: age (years) × AST (IU/L)/(platelet count (10^9^/L) × ALT (IU/L)^1/2^ [15].

### Magnetic resonance imaging

**Fig 1:**
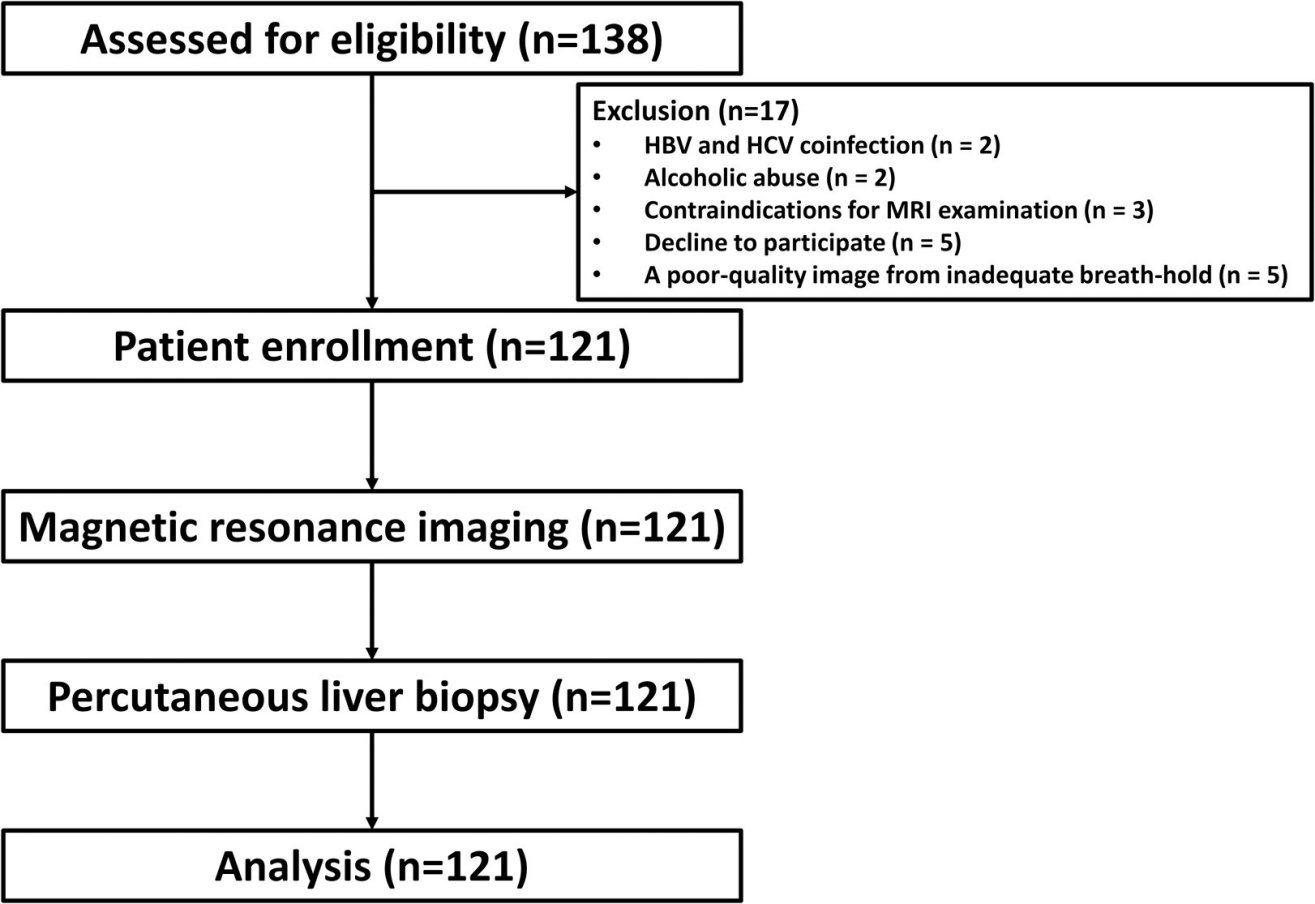
Flow diagram of the trial.

All MR images were performed on a 3-T scanner (Ingenia; Philips Medical Systems, Best, The Netherlands) before the liver biopsy examination on the same day (Fig 1). DWI was acquired using a single-shot, breath-hold, echo-planar imaging fat-suppressed sequence in the 2D axial view covering the whole liver. The acquisition was performed using the following parameters: repetition time (TR)/echo time (TE), 1,600–2,000/51-60 msec; slice thickness, 5 mm; interslice gap, 1 mm; field of view, up to 400 mm with 80% rectangular field of view; matrix size, 256×256; parallel imaging factor, 2 with 2 averages; *b*-values of 0, 400, 600, 800, 1,000, and 1,200 s/mm^2^; and tridirectional diffusion gradients with trace image used for analysis.

Automatic voxel-by-voxel analysis on a workstation (Easy Vision Workstation, release 5.1; Philips Medical System) was used to obtain ADC maps for the *b*-values of 400, 600, 800, 1,000, and 1,200 s/mm^2^. All image analysis was done by a single experienced radiologist who was blinded to all clinical data. ADCs were measured in the lateral and medial segments of the left lobe and the anterior and posterior segments of the right lobe with circular regions of interest (ROIs) away from normal intrahepatic vasculature and at least 1 cm apart from the Glisson’s capsule. The final ADC per subject used for analysis was the average of the ROI measures. The spleen was selected to serve as a reference within each patient. For the ADC measurements of the spleen, the greatest diameter was chosen to place the ROIs, avoiding vessels and spleen capsule. Normalized liver ADC was calculated as the ratio of liver ADC to spleen ADC.

### Histological assessment

The percutaneous liver biopsy was performed using Menghini’s technique with a 1.6 mm needle (Hepafix, Braun, Melsungen, Germany). Tissue samples were fixed in formalin, paraffin-embedded, and subjected to standard techniques for Hematoxylin and Eosin, Masson trichrome, and Perls’staining. All specimens were analyzed by a single hepatopathologist who was unaware of the clinical data. Histological staging of fibrosis was identified based on the METAVIR scoring system and divided into the following five stages: F0, no fibrosis; F1, portal fibrosis without septa; F2, portal fibrosis with few septa; F3, bridging septa between central and portal veins; and F4, cirrhosis [16]. Significant fibrosis was defined as F2 or higher stages and advanced fibrosis was defined as F3 or higher stages. Necroinflammatory activity and hepatic steatosis were graded according to the proposal by Kleiner *et al*. [17]. Hepatic iron content was evaluated according to Deugnier’s original score and was reported with the mean score of the sample ranging from 0 to 33 [18].

### Statistical analysis

Based on previous studies of DWI for determining liver fibrosis stage with histology as a reference in a variety of etiologies [19], we assumed the AUROC of DWI to be 0.80 for a diagnosis of cirrhosis in patients with chronic viral hepatitis. A minimum sample size of 120 was estimated to achieve a power of 0.90 with an alpha of 0.05.

Descriptive statistics were used to determine the characteristics of the study population. Standard parametric and nonparametric statistics were used for the comparison of variables. The Spearman’s rank correlation test was used to assess the relationships of ADC values with the severity of histological features. The logistic regression model was used to identify factors independently associated with discordance. The area under the receiver-operating characteristics (AUROC) curve was constructed to evaluate the overall accuracy of DWI and was compared between the ADCs at different *b*-values with the DeLong test. The optimal cutoffs of diffusion parameters for F2, F3, and F4 diseases were chosen at points with the highest Youden’s index. Data analysis was executed with the SPSS software package version 18.0 (SPSS Inc., Chicago, IL).

## Results

### Patient characteristics

Between June 2014 to April 2016, 138 consecutive naïve patients with chronic viral hepatitis were enrolled, but 17 patients were excluded from this study due to HBV and HCV coinfection (n = 2), alcoholic abuse (n = 2), contraindications for MRI examination (n = 3), decline to participate (n = 5), and a poor-quality image from inadequate breath-hold (n = 5) as shown in Fig 1. Therefore, the cohort consisted of 121 patients, including 61 HBV-infected patients and 60 HCV-infected patients. The clinical, laboratory, and histologic characteristics of the subjects according to the viral etiology are summarized in Table 1. The mean age of the cohort was 48.4±10 years, and 43% were male. The mean body mass index (BMI) was 24.2±4.0 kg/m^2^, with the prevalence of diabetes of 7.5%. Moderate necroinflammation was observed in 43 patients (36%), and steatosis was present in 57 patients (47%). According to the METAVIR scoring system, five patients (4%) were staged as F0, 50 patients (41%) as F1, 42 patients (35%) as F2, 16 patients (13%) as F3, and eight patients (7%) as F4. The histological feature of iron deposits was observed in 93 (77%).

**Table 1:** Demographic and clinical characteristics of the study population.

Variables	All patients	Chronic hepatitis B	Chronic hepatitis C	p-value
	(n = 121)	(n = 61)	(n = 60)	
Age (years)	48.4±10.4	45.8±9.8	51.0±10.4	0.007
Male sex, n (%)	52 (43%)	26 (43%)	26 (43%)	0.937
Body mass index (kg/m^2^)				
<25 kg/m^2^	78 (65%)	39 (64%)	39 (65%)	0.989
25–30 kg/m^2^	33 (27%)	17 (28%)	16 (27%)	
>30 kg/m^2^	10 (8%)	5 (8%)	5 (8%)	
Waist circumference (cm)	86.7±10.4	86.9±10.6	86.5±10.3	0.981
Fasting glucose (g/dL)	104±23	105±26	103±20	0.944
Total cholesterol (g/dL)	186±43	195±44	177±40	0.001
Triglycerides (g/dL)	96±45	98±52	93±36	0.878
AST (IU/L)	50±35	35±18	65±41	<0.001
ALT (IU/L)	58±45	43±33	74±51	<0.001
Platelet count (x 10^9^/L)	210±57	223±55	197±57	0.010
FIB-4 index	1.76±1.38	1.31±1.06	2.23±1.50	<0.001
Diabetes	9 (7.5%)	5 (8%)	4 (7%)	0.881
Hypertension	36 (30%)	17 (28%)	19 (32%)	0.648
METAVIR fibrosis stage				
F0	5 (4%)	5 (8%)	0	0.011
F1	50 (41%)	29 (48%)	21 (35%)	
F2	42 (35%)	21 (34%)	21 (35%)	
F3	16 (13%)	5 (8%)	11 (18%)	
F4	8 (7%)	1 (2%)	7 (12%)	
Hepatic inflammation				
A0	4 (3%)	4 (7%)	0	<0.001
A1	74 (61%)	46 (75%)	28 (47%)	
A2	43 (36%)	11 (18%)	32 (53%)	
A3	0	0	0	
Steatosis grade				
<5% steatosis	64 (53%)	30 (49%)	34 (56%)	0.520
5–30% steatosis	45 (37%)	24 (39%)	21 (35%)	
30–60% steatosis	11 (9%)	7 (12%)	4 (7%)	
>60% steatosis	1 (1%)	0	1 (2%)	
Total hepatic iron index	6.0 (1.0–9.25)	7.0 (2.75–9.25)	2.5 (0.5–9.5)	0.071

HCV-infected patients were older with higher aminotransferase levels and lower platelet counts, contributing to a higher FIB-4 score than HBV-infected patients (Table 1). The proportions of patients with moderate necroinflammation and advanced fibrosis/cirrhosis were significantly higher in the HCV group. Otherwise, gender, BMI, degree of steatosis distributions, and hepatic iron index were similar between both two groups.

### The relationship between histological features and apparent diffusion coefficient

There was a significant positive correlation between spleen ADC and fibrosis stage, the *r* values ranging from 0.190 for the *b*-value of 1,200 s/mm^2^ to 0.221 for the *b*-value of 400 s/mm^2^ (Table 2). There was a significant inverse correlation between liver ADC and fibrosis stage, with the *r* values of −0.255 for the *b*-value of 1,000 s/mm^2^ and −0.179 for the *b*-value of 1,200 s/mm^2^. Similarly, there was a significant decrease in normalized liver ADC at all b-values with an increasing degree of liver fibrosis, with the *r* values ranging from −0.213 for the *b*-value of 600 s/mm^2^ to −0.292 for the *b*-value of 800 s/mm^2^. The best correlation was observed for liver ADC at the *b*-value of 1,000 s/mm^2^ (*r* = –0.255, p = 0.005) and for normalized liver ADC at the *b*-value of 800 s/mm^2^ (*r* = –0.292, p = 0.001).

**Table 2:** Distribution of liver and normalized liver apparent diffusion coefficients value (× 10^−3^ mm^2^/s) stratified by fibrosis stage.

b Value (s/mm^2^)	METAVIR Fibrosis Stage						
	F0	F1	F2	F3	F4	*r*	*p*-Value*
**Liver ADC**							
400	1.50 ± 0.14	1.47 ± 0.24	1.41 ± 0.17	1.47 ± 0.14	1.38 ± 0.13	-0.086	0.350
600	1.42 ± 0.16	1.25 ± 0.14	1.28 ± 0.12	1.23 ± 0.12	1.20 ± 0.10	-0.173	0.058
800	1.20 ± 0.12	1.17 ± 0.11	1.14 ± 0.10	1.15 ± 0.11	1.11 ± 0.06	-0.174	0.057
1,000	1.16 ± 0.07	1.09 ± 0.10	1.08 ± 0.10	1.03 ± 0.07	1.03 ± 0.05	-0.255	***0*.*005***
1,200	1.06 ± 0.07	1.03 ± 0.10	1.04 ± 0.08	0.99 ± 0.10	0.98 ± 0.09	-0.179	***0*.*049***
**Spleen ADC**							
400	1.21 ± 0.12	1.25 ± 0.25	1.36 ± 0.30	1.33 ± 0.33	1.48 ± 0.23	0.221	***0*.*015***
600	1.17 ± 0.12	1.10 ± 0.18	1.10 ± 0.20	1.13 ± 0.21	1.30 ± 0.24	0.151	0.099
800	0.98 ± 0.04	0.99 ± 0.16	1.00 ± 0.18	1.03 ± 0.17	1.15 ± 0.16	0.201	***0*.*027***
1,000	0.92 ± 0.14	0.92 ± 0.13	0.93 ± 0.12	0.91 ± 0.15	1.08 ± 0.18	0.177	0.053
1,200	0.88 ± 0.08	0.86 ± 0.13	0.87 ± 0.13	0.88 ± 0.10	1.00 ± 0.20	0.190	***0*.*037***
**Normalized Liver ADC**							
400	1.25 ± 0.18	1.21 ± 0.26	1.12 ± 0.19	1.13 ± 0.19	0.94 ± 0.11	-0.285	***0*.*002***
600	1.22 ± 0.16	1.16 ± 0.20	1.20 ± 0.23	1.11 ± 0.17	0.94 ± 0.13	-0.213	***0*.*019***
800	1.23 ± 0.14	1.20 ± 0.18	1.17 ± 0.18	1.13 ± 0.17	0.97 ± 0.11	-0.292	***0*.*001***
1,000	1.27 ± 0.15	1.20 ± 0.17	1.18 ± 0.14	1.17 ± 0.26	0.97 ± 0.13	-0.273	***0*.*002***
1,200	1.21 ± 0.07	1.22 ± 0.19	1.21 ± 0.20	1.14 ± 0.08	1.00 ± 0.15	-0.271	***0*.*003***

*Spearman’s rank correlation coefficient was used to examine correlations of ADC with the progression of fibrosis.

The liver ADC at all b-values decreased as the necroinflammatory activity increased, the r values ranging from −0.226 for the b-value of 1,200 s/mm^2^ to −0.384 for the b-value of 800 s/mm^2^. There was a significant negative correlation between the degree of steatosis and liver ADC at b-values of 800 s/mm^2^ (r = -0.189, p = 0.038), 1,000 s/mm^2^ (r = -0.181, p = 0.048), and 1,200 s/mm^2^ (r = -0.299, p<0.001). A significant correlation between fibrosis stage and liver ADC value disappeared after adjusting for necroinflammation and steatosis. The normalized liver ADC at all b-values decreased with an increasing degree of necroinflammation, the r values ranging from −0.172 for a b-value of 400 s/mm^2^ to −0.249 for a b-value of 1,200 s/mm^2^. In contrast, normalized liver ADC at any b-values was not associated with the degree of steatosis. The significant negative correlation between fibrosis stage and normalized liver ADC at b-values of 400, 800, and 1,000 s/mm^2^ remained after adjusting for necroinflammatory activity (p<0.05).

### The performance of normalized liver ADC for staging liver fibrosis

The normalized liver ADC values at the *b*-value of 800 s/mm^2^ among patients with F0-1, F2, F3, and F4 diseases were 1.21±0.17, 1.17±0.18, 1.13±0.17, and 0.97±0.11×10^−3^ mm^2^/s, respectively (p = 0.005 by analysis of variance). Overall, the accuracy of normalized liver ADC for detecting significant fibrosis was poor with the AUROCs of 0.548 to 0.622, and for detecting advanced fibrosis was fair with the AUROCs of 0.631 to 0.709, while it was good for determining cirrhosis with the AUROCs of 0.832 to 0.853 (Table 3). In an additional subgroup analysis of the etiology of viral infection, no significant differences between the HBV and the HCV groups were observed in AUROCs of normalized liver ADC at the *b*-value of 800 s/mm^2^ for detecting significant fibrosis (0.632 versus 0.549, p = 0.432) and advanced fibrosis (0.636 versus 0.706, p = 0.593).

**Table 3:** Performance of normalized liver ADC at the different b-values for detecting each fibrosis stage.

b-Value (s/mm^2^)	AUROC	Cut-off value	Sensitivity	Specificity	PPV	NPV	LR+	LR-
	(95% CI)	(×10^−3^ mm^2^/s)	(95% CI)	(95% CI)	(95% CI)	(95% CI)	(95% CI)	(95% CI)
**Fibrosis stage ≥2**								
400	0.622 (0.529–0.708)	≤1.01	33.3 (22.2–46.0)	87.3 (75.5–94.7)	75.9 (59.2–87.2)	52.2 (47.2–57.1)	2.62 (1.2–5.7)	0.76 (0.6–0.9)
600	0.548 (0.455–0.639)	≤0.99	27.3 (17.0–39.6)	89.1 (77.8–95.9)	75.0 (56.1–87.5)	50.5 (46.2–54.9)	2.50 (1.1–5.9)	0.82 (0.7–1.0)
800	0.603 (0.510–0.691)	≤1.13	53.0 (40.3–65.4)	65.5 (51.4–77.8)	64.8 (54.5–73.9)	53.7 (45.7–61.5)	1.54 (1.0–2.4)	0.72 (0.5–1.0)
1,000	0.594 (0.501–0.683)	≤1.04	34.9 (23.5–47.6)	87.3 (75.5–94.7)	76.7 (60.4–87.6)	52.7 (47.7–57.8)	2.74 (1.3–5.9)	0.75 (0.6–0.9)
1,200	0.599 (0.506–0.687)	≤1.19	65.2 (52.4–76.5)	54.6 (40.6–68.0)	63.2 (55.1–70.7)	56.6 (46.4–66.2)	1.43 (1.0–2.0)	0.64 (0.4–1.0)
**Fibrosis stage ≥3**								
400	0.631 (0.539–0.717)	≤0.95	37.5 (18.8–59.4)	89.7 (81.9–94.9)	47.4 (29.2–66.3)	85.3 (80.9–88.8)	3.64 (1.7–8.0)	0.70 (0.5–1.0)
600	0.695 (0.605–0.775)	≤1.15	79.2 (57.8–92.9)	52.6 (42.2–62.8)	29.2 (23.5–35.6)	91.1 (82.1–95.8)	1.67 (1.2–2.2)	0.40 (0.2–0.9)
800	0.704 (0.614–0.783)	≤1.05	58.3 (36.6–77.9)	79.4 (70.0–86.9)	41.2 (29.5–54.0)	88.5 (82.6–92.6)	2.83 (1.7–4.7)	0.52 (0.3–0.9)
1,000	0.691 (0.601–0.772)	≤1.04	58.3 (36.6–77.9)	81.4 (72.3–88.6)	43.8 (31.3–57.1)	88.8 (83.0–92.8)	3.14 (1.8–5.4)	0.51 (0.3–0.8)
1,200	0.709 (0.619–0.788)	≤1.18	83.3 (62.6–95.3)	56.7 (46.3–66.7)	32.3 (26.3–38.9)	93.2 (84.7–97.2)	1.92 (1.4–2.6)	0.29 (0.1–0.7)
**Fibrosis stage 4**								
400	0.832 (0.753–0.894)	≤1.08	87.5 (47.3–99.7)	58.4 (48.8–67.6)	13.0 (9.6–17.3)	98.5 (91.3–99.8)	2.10 (1.5–3.0)	0.21 (0.03–1.3)
600	0.847 (0.771–0.906)	≤0.97	75.0 (34.9–96.8)	88.5 (81.1–93.7)	31.6 (19.4–46.9)	98.0 (93.8–99.4)	6.52 (3.4–12.5)	0.28 (0.08–0.9)
800	0.847 (0.771–0.906)	≤1.02	87.5 (47.3–99.7)	81.4 (73.0–88.1)	25.0 (17.3–34.7)	98.9 (93.6–99.8)	4.71 (3.0–7.5)	0.15 (0.02–1.0)
1,000	0.853 (0.777–0.911)	≤1.04	87.5 (47.3–99.7)	77.9 (69.1–85.1)	21.9 (15.4–30.2)	98.9 (93.3–99.8)	3.95 (2.6–6.1)	0.16 (0.03–1.0)
1,200	0.834 (0.756–0.895)	≤1.17	87.5 (47.3–99.7)	53.1 (43.5–62.5)	11.7 (8.7–15.5)	98.4 (90.5–99.7)	1.87 (1.3–2.6)	0.24 (0.04–1.5)

Abbreviation: ADC, Apparent diffusion coefficient; LR-, negative likelihood ratio; LR+, positive likelihood ratio; NPV, negative predictive value; PPV, positive predictive value.

For detecting significant fibrosis, the AUROC of normalized liver ADC at a *b*-value of 800 s/mm^2^ was comparable with those of normalized liver ADC with other *b*-values. The optimal cutoff for significant fibrosis was ≤1.13×10^−3^ mm^2^/s by normalized liver ADC at a *b*-value of 800 s/mm^2^ (Table 3). The positive predictive value (PPV) to confirm significant fibrosis was 64.8% (95% CI, 54.5%-73.9%).

For detecting advanced fibrosis, the AUROC of normalized liver ADC at a *b*-value of 800 s/mm^2^ was comparable with normalized liver ADC with other *b*-values. The optimal cutoff for advanced fibrosis was ≤1.05×10^−3^ mm^2^/s by normalized liver ADC at a *b*-value of 800 s/mm^2^ (Table 3). The negative predictive value (NPV) to exclude advanced fibrosis was 88.5% (95% CI, 82.6%-92.6%).

For detecting cirrhosis, the AUROC of normalized liver ADC at a *b*-value of 800 s/mm^2^ was comparable with normalized liver ADC with other *b*-values. The optimal cutoff for cirrhosis was ≤1.02×10^−3^ mm^2^/s by normalized liver ADC at a *b*-value of 800 s/mm^2^ (Table 3). The NPV to exclude cirrhosis was 98.9% (95% CI, 93.6%-99.8%).

### Prevalence and risk factors of discordance

Discordance ≥2 fibrosis stages between DWI and histology were observed in 25 (20.6%) of the patients assessed with normalized liver ADC at a *b*-value of 800 s/mm^2^ (Table 4). Fibrosis estimated by normalized liver ADC (F3-F4) exceeded liver biopsy (F0-F2) in 72% (n = 18) of discordant cases. By univariate analysis, elevated ALT and shorter liver biopsy lengths were associated with discordance. Discordance occurred in 16 of 54 (29.6%) patients with liver biopsy lengths shorter than 15 mm, compared with 9 of 67 (13.4%) patients with liver biopsy lengths 15 mm or higher (p = 0.029). Age, gender, BMI, waist circumference, the etiology of viral infection, the degree of steatosis and necroinflammation, total hepatic iron index, and lower fibrosis stage were not associated with discordance. By multivariate analysis, only elevated ALT (odds ratio, 1.53 per times the upper limit of normal; 95% CI 1.10–2.14; p = 0.011) remained an independent risk factor associated with discordance.

**Table 4:** Distribution of fibrosis according to liver histology and as estimated by diffuse-weighted magnetic resonance imaging.

Fibrosis according to liver histology	Fibrosis according to normalized liver ADC*			
	F0-1 (n = 68)	F2 (n = 21)	F3 (n = 6)	F4 (n = 26)
**F0-1** (n = 55)	36	8	2	9
**F2** (n = 42)	25	9	1	7
**F3** (n = 16)	6	4	2	4
**F4** (n = 8)	1	0	1	6

Discordance ≥2 stages between biopsy and diffuse-weighted magnetic resonance imaging indicated by grey squares.

Abbreviation: ADC, Apparent diffusion coefficient

* Normalized liver apparent diffusion coefficients were measured with a b-value of 800 s/mm^2^

### Clinical use of DWI for predicting cirrhosis

Two strategies using DWI with normalized liver ADC at a *b*-value of 800 s/mm^2^ to diagnose and exclude cirrhosis in patients with chronic viral hepatitis were evaluated: (1) use an optimal cut-off of normalized liver ADC for all patients; (2) use a sequential approach with the FIB-4 index followed by DWI only for patients who have indeterminate to high probabilities of cirrhosis with FIB-4 score more than 1.45.

**Fig 2:**
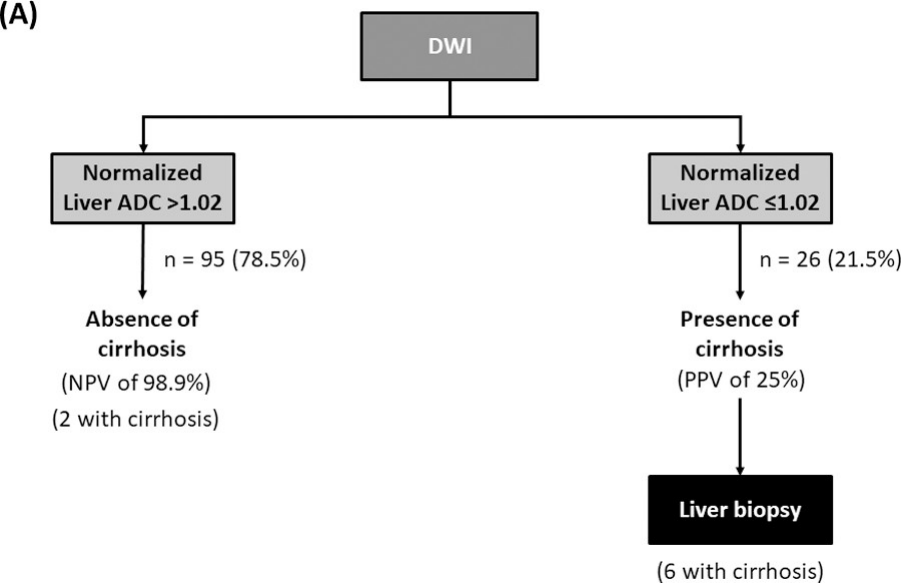
Clinical use of diffusion-weighted magnetic resonance imaging (DWI) with normalized liver apparent diffusion coefficient.

Using DWI with normalized liver ADC >1.02×10^−3^ mm^2^/s for excluding cirrhosis, two (1.7%) patients with cirrhosis would be missed. If liver biopsies were reserved for patients with normalized liver ADC ≤1.02×10^−3^ mm^2^/s, 26 (21.5%) patients would require the procedure to establish cirrhosis (Fig 2).

**Fig 3:**
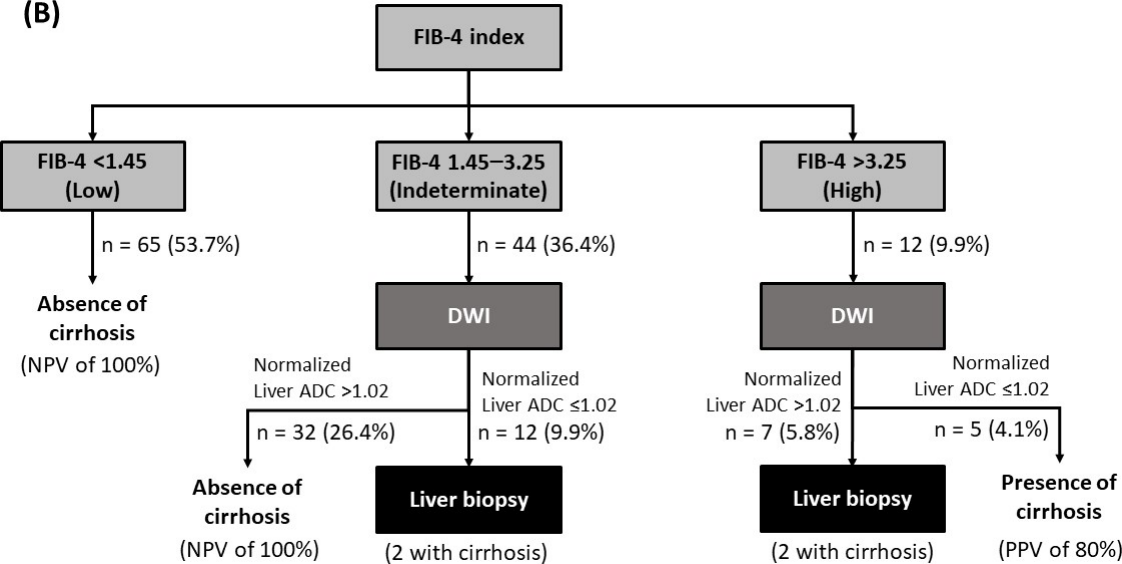
A sequential approach of the FIB-4 index followed by diffusion-weighted magnetic resonance imaging (DWI) with normalized liver apparent diffusion coefficient.

Because of the considerably low PPV of DWI, we develop an algorithm to improve the post-test probability and reduce unnecessary MRI examination by incorporating the FIB-4 index in the clinical setting with a low prevalence of cirrhosis. When this approach was used, cirrhosis was excluded in 65 (53.7%) patients with FIB-4 score <1.45, as shown in Fig 3. Of patients with FIB-4 score >3.25 who had a high probability of cirrhosis, the PPV of normalized liver ADC ≤1.02×10^−3^ mm^2^/s for the diagnosis of cirrhosis was improved to 80%. For patients with FIB-4 score between 1.45 and 3.25, normalized liver ADC >1.02×10^−3^ mm^2^/s yielded an NPV of 100% for excluding cirrhosis. Liver biopsies would be reserved for patients with discordant results as follows: patients with FIB-4 score between 1.45 and 3.25 and normalized liver ADC ≤1.02×10^−3^ mm^2^/s, and those with FIB score >3.25 and normalized liver ADC >1.02×10^−3^ mm^2^/s. Thus, only 15.7% of patients would require a liver biopsy. This sequential strategy can reduce unnecessary MRI examinations by 53.7%.

## Discussion

In this prospective cohort of patients with chronic viral hepatitis, we performed DWI using different *b*-values to obtain liver ADC and normalized liver ADC using the spleen as a reference organ. The normalized liver ADC values at different *b*-values showed significant correlations with the fibrosis stage. Normalized liver ADC had high accuracy in the detection of cirrhosis. Most discordances between normalized liver ADC and histology occurred in patients with elevated ALT. In the clinical setting with a low prevalence of cirrhosis, the sequential use of the FIB-4 index, followed by DWI, appears to be an optimal approach for cirrhosis.

DWI is an attractive technique for the quantitative assessment of tissue diffusivity without using a special device. The ADC valued by DWI is an indicator of the movement of water molecules within the tissue and provides useful information about slow-perfusion, inflammation, and local cell breakdown [20]. Several studies showed that water diffusion might be diminished by extracellular collagen fibers, the main component of liver fibrosis; consequently, reduced ADC values have been reported for liver fibrosis [8, 10, 11, 13, 21–24]. These findings suggest that DWI could be a useful imaging technique for assessing liver fibrosis in chronic liver disease. Previous studies found a significant negative correlation between ADC values and the fibrosis stage [9, 10, 12]. However, these findings remain speculative because of the limited number of patients involved in these studies. Extending previous results [9, 22], our study in a larger cohort showed that ADC values were lower in cases with more advanced stage of fibrosis, and its discriminating abilities between patients with different stages of liver fibrosis were statistically significant. The decreased ADC values could partly be due to restricted water diffusion in the presence of increased fibrotic tissue in the liver [25].

Differences in imaging quality and acquisition parameters make it difficult to compare the results from these studies. In this regard, the choice of the *b*-value for ADC plays a critical role. Recent studies have often selected *b*-values of 500 s/mm^2^ or larger to quantify liver fibrosis [8, 13, 23, 24]. Thus, the liver ADC values were of smaller distribution, and the perfusion component was minimized. In agreement with these studies, our results from a larger sample of chronic viral hepatitis demonstrated that the liver ADC values acquired from *b*-values of at least 1,000 s/mm^2^ showed a significant correlation with the liver fibrosis stage. In this study, we clearly showed that necroinflammation and steatosis influenced the liver ADC values, indicating that liver ADC is not attributable to fibrosis alone. Hence, liver ADC value has limited usefulness for assessing liver fibrosis in cases with a significant degree of necroinflammation and steatosis.

The accuracy of liver ADC for the quantification of fibrosis also is conditioned by variability in the measurement process. Normalization of liver ADC using a reference organ that remains relatively constant across patients or imaging platforms may reduce variability in ADC calculations. A study by Do *et al*. [13] showed that normalized liver ADC using the spleen as the reference organ attenuated liver ADC variability and improved accuracy for detecting liver fibrosis. Our analysis showed an improved correlation of the liver fibrosis stage and normalized liver ADC using the spleen as the reference, especially with a *b*-value of 800 s/mm^2^. We found that the normalization of ADC was better than standard ADC measurement in the differentiation of each fibrosis stage. Although normalized liver ADC could be influenced by necroinflammation, the extent of hepatic necroinflammation has no major impact on the use of normalized liver ADC for assessing liver fibrosis. Likewise, steatosis did not affect normalized liver ADC value. Based on these data, the normalized liver ADC is of value in liver fibrosis staging. The ability of DWI to distinguish advanced fibrosis and cirrhosis was improved in our cohort by normalizing liver ADC with spleen ADC obtained at all *b*-values from 400 to 1,200 s/mm^2^. The absolute spleen ADC increased slightly with the increased liver fibrosis stage, especially for cirrhosis. This data suggest that normalized liver ADC may serve as a surrogate for the dynamic component of portal hypertension, resulting in better performance in discriminating the presence of liver cirrhosis. Interestingly, the performance of normalized liver ADC with a higher *b*-value did not show any improvement for the detection of cirrhosis. This finding may be explained by the drawbacks of DWI (i.e., sensitivity to motion artifacts and noise at high *b-*values) [26]. Moreover, the degree of signal attenuation of the liver with increasing *b*-value is nonlinear due to microcapillary perfusion [25, 27].

DWI has been validated in chronic viral hepatitis, autoimmune liver disease, alcohol-associated liver disease, and nonalcoholic fatty liver disease [8, 13, 24, 28]. According to a meta-analysis of ten studies evaluating DWI for the staging of liver fibrosis, the pooled estimates of sensitivity and specificity are 77% and 78%, respectively, for significant fibrosis and 72% and 84%, respectively, for advanced fibrosis [20]. Our study showed that the accuracy of DWI was less than initially reported. However, DWI assessed with normalized liver ADC at different *b*-values had high diagnostic accuracies for detecting cirrhosis with AUROCs of 0.832 to 0.853. At the cutoff of 1.02×10^−3^ mm^2^/s by normalized liver ADC at a *b*-value of 800 s/mm^2^, the NPV to exclude cirrhosis was 99%. The high accuracy of DWI in the discrimination of cirrhosis is critical because those patients should be screened for portal hypertension and hepatocellular carcinoma. It is worth noting that DWI as an adjunct to routine MRI protocol is capable of providing information about background liver cirrhosis in patients with nodular liver lesions, yielding a correct diagnosis of the regenerative nodule, dysplastic nodule, and hepatocellular carcinoma.

In our study of chronic viral hepatitis, discordance between DWI and histology occurs more commonly if the ALT level is high. This finding suggests that discordance was attributable to overestimating by DWI as a result of hepatic inflammation. ALT level in subjects with chronic viral hepatitis mainly reflects inflammation or necrosis of the whole liver. Consistent with a study by Taouli *et al*. [8], we found that the ADC values significantly correlated with necroinflammatory activity, suggesting that necroinflammation can lead to misclassified liver fibrosis stage with DWI. However, this histological feature was not associated with discordance. One possible explanation of the phenomenon is that an evaluation of disease severity with liver biopsy might not represent liver tissue alteration across the entire organ. Thus, the discordance between DWI and histology could result from sampling errors, especially with short biopsy samples. This is supported by Poynard *et al*. reporting that specimens longer than 15 mm provided a better correlation of liver histology with fibrosis biomarkers than smaller samples [29].

Although DWI measured with normalized liver ADC showed a high NPV for cirrhosis, it does not adequately rule-in cirrhosis. Several studies showed that the diagnostic performance of imaging modalities, i.e., ultrasound-based elastography for advanced fibrosis/cirrhosis, increased when combined with the FIB-4 index, which is the most widely used formula to screen for advanced fibrosis [30–32]. However, measuring liver stiffness requires special equipment that is not available in many institutions. Hence, we propose an algorithm to improve DWI’s performance for cirrhosis diagnosis by incorporating the FIB-4 index in the clinical setting with a low prevalence of cirrhosis. The sequential approach using the FIB-4 index followed by DWI for patients with indeterminate and high FIB-4 is better than using DWI for all patients. With this approach, the FIB-4 score <1.45 would exclude cirrhosis. Importantly, a sequential strategy using FIB-4 score >3.25 and normalized liver ADC ≤1.02×10^−3^ mm^2^/s improved PPV of DWI without much effect on misclassification. Patients with discordant results following the sequential approach may undergo a liver biopsy for the definite diagnosis or be followed up with repeated DWI annually, depending on local practice.

Our study has some limitations. First, a liver needle biopsy was used as the gold standard. However, sampling bias could not be excluded. Second, only 7% of our cohort had liver cirrhosis. Thus, we cannot exclude that this distribution may have improved NPVs in excluding cirrhosis.

In conclusion, normalized liver ADC using the spleen as a reference organ holds promise as a noninvasive imaging technique for assessing cirrhosis in patients with chronic viral hepatitis. DWI can be routinely used as part of the comprehensive MRI protocol to detect cirrhosis while monitoring patients with chronic viral hepatitis. The utility of ADC normalization in patients with other etiologies remains to be explored in future research.

## Supporting information

S1 Data Baseline characteristics of the study population. (XLSX)

S2 Data (XLSX)

S3 Data (DOCX)

S1 Checklist TREND statement checklist. (PDF)

## Abbreviations

ADC: apparent diffusion coefficient
ALT: alanine aminotransferase
AST: aspartate aminotransferase
AUROC: the area under the receiver-operating characteristics
BMI: body mass index
DWI: diffuse weighted imaging
FIB-4: fibrosis-4
HBV: hepatitis B virus
HCV: hepatitis C virus
MRI: magnetic resonance imaging
NPV: negative predictive value
PPV: positive predictive value
ROI: region of interest
TE: echo time
TR: repetition time
