# Supplementary material for: Diffusion-weighted magnetic resonance imaging for the assessment of liver fibrosis in chronic viral hepatitis
Source: PLoS One. 2021 Mar 4;16(3):e0248024. doi: 10.1371/journal.pone.0248024 (PMC7932524; doi:10.1371/journal.pone.0248024)
Supplement: S3 Data — (DOCX) [file pone.0248024.s003.docx]

**Diffusion-weighted Imaging Magnetic Resonance for Assessing Liver Fibrosis in Patients with Chronic Viral Hepatitis**

ClinicalTrials.gov Identifier: NCT02682108

Other Study ID Numbers: Si237/2014

Phunchai Charatcharoenwitthaya, Mahidol University

**Brief Summary:**

Several noninvasive radiological techniques have been investigated for the diagnosis of liver fibrosis and cirrhosis among patients with chronic infection with hepatitis B virus or hepatitis C virus. Diffusion-weighted magnetic resonance imaging (DW-MRI) is a particularly appealing method for the diagnosis of liver fibrosis. The aims of this study are to evaluate the accuracy of DW-MRI in patients with chronic viral hepatitis for determining the stage of liver fibrosis.

**Detailed Description:**

Among patients with chronic infection with hepatitis B virus or hepatitis C virus, evaluation of the stage of liver fibrosis is of major importance for determining prognosis and therapeutic decisions. Liver biopsy is a costly and invasive technique with associated mortality and morbidity. A typical biopsy fragment represents only 1/50,000 of the organ. Several noninvasive radiological techniques have been investigated for the diagnosis of liver fibrosis and cirrhosis. Diffusion-weighted magnetic resonance imaging (DW-MRI) is a particularly appealing method for the diagnosis of liver fibrosis. Because it is easy to implement, non-operator dependent, and process without the need for contrast agents. However, preliminary studies on small numbers of patients in which various hardware and sequencing profiles were used have reported inconsistent results for staging liver fibrosis with DW-MRI. The aims of this study are to evaluate correlation between stage of hepatic fibrosis and liver apparent diffusion coefficient (ADC) and normalized liver ADC with spleen assessed by DW-MRI in patients with chronic viral hepatitis B or C. Also, this study aim to evaluate factors that influence liver ADC and normalized liver ADC with spleen value for predicting the stage of liver fibrosis as well as to estimate the optimal cutoff values of DW-MRI for determining significant liver fibrosis (fibrosis stage ≥2) and advanced fibrosis (fibrosis stage ≥3).

**Study Design**

Study Type: Interventional (Clinical Trial)

Actual Enrollment: 121 participants

Allocation: N/A

Intervention Model: Single Group Assignment

Masking: None (Open Label)

Primary Purpose: Diagnostic

**Eligibility Criteria**

Ages Eligible for Study: 18 Years to 80 Years (Adult, Older Adult)

Sexes Eligible for Study: All

Accepts Healthy Volunteers: No

***Inclusion Criteria:***

All naive patients with chronic hepatitis B or C infection who undergo liver biopsy examination for evaluating candidates for antiviral therapy will be invited to participate into this study

***Exclusion Criteria:***

Other cause of chronic liver disease

Contraindication for liver biopsy

Contraindication for magnetic resonance imaging

Contacts and Locations

**Locations**

Faculty of Medicine Siriraj Hospital, Bangkoknoi, Bangkok, Thailand, 10700

Sponsors and Collaborators: Faculty of Medicine Siriraj Hospital

Principal Investigator: Phunchai Charatcharoenwitthaya, MD

## Clinical and biological parameters

## A comprehensive clinical assessment will be performed. On the day of liver biopsy, a fasting venous blood sample will be collected from all patients for aspartate aminotransferase (AST), alanine aminotransferase (ALT), complete blood count, glucose, total cholesterol, and triglycerides.

## Magnetic resonance imaging

## All MR images will be performed on a 3-T scanner (Ingenia; Philips Medical Systems, Best, The Netherlands) before the liver biopsy examination, on the same day. DWI will be acquired using a single-shot, breath-hold, echo-planar imaging fat-suppressed sequence in the 2D axial view covering the whole liver. The acquisition will be performed using the following parameters: repetition time (TR)/echo time (TE), 1,600-2,000/51-60 msec; slice thickness, 5 mm; interslice gap, 1 mm; field of view, up to 400 mm with 80% rectangular field of view; matrix size, 256×256; parallel imaging factor, 2 with 2 averages; *b*-values of 0, 400, 600, 800, 1,000, and 1,200 s/mm^2^; and tridirectional diffusion gradients with trace image used for analysis.

## Automatic voxel-by-voxel analysis on a workstation (Easy Vision Workstation, release 5.1; Philips Medical System) will be used to obtain ADC maps for the *b*-values of 400, 600, 800, 1,000, and 1,200 s/mm^2^. All image analysis will be done by a single experienced radiologist who is blinded to all clinical data. ADCs will be measured in the lateral and medial segments of the left lobe and the anterior and posterior segments of the right lobe with circular regions of interest (ROIs) away from normal intrahepatic vasculature and at least 1 cm apart from the Glisson’s capsule. The final ADC per subject used for analysis is the average of the ROI measures. The spleen will be selected to serve as a reference within each patient. For the ADC measurements of the spleen, the greatest diameter will be chosen to place the ROIs, avoiding vessels and spleen capsule. Normalized liver ADC is calculated as the ratio of liver ADC to spleen ADC.

**Histological assessment**

The percutaneous liver biopsy will be performed using Menghini’s technique with a 1.6 mm needle (Hepafix, Braun, Melsungen, Germany). The liver specimens will be placed in a container, fixed in formalin, paraffin-embedded, and subjected to standard techniques for Hematoxylin and Eosin, Masson trichrome, and Perls’staining. Liver histology will be assessed by an experienced pathologist who is not aware of the clinical data. Histologic staging of fibrosis is identified based on the METAVIR scoring system and divided into the following five stages: F0, no fibrosis; F1, portal fibrosis without septa; F2, portal fibrosis with few septa; F3, bridging septa between central and portal veins; and F4, cirrhosis.[[10](#_ENREF_10)] We considered grades of F2 or higher as significant fibrosis and grades of F3 or higher as advanced fibrosis. Necroinflammatory activity and steatosis are graded according to the proposal by Kleiner *et al*. Hepatic iron content is evaluated according to Deugnier’s original score and will be reported with the mean score of the sample ranging from 0 to 33.

**Outcome Measures**

***Primary Outcome Measures:***

The correlation coefficient of the stage of hepatic fibrosis and liver apparent diffusion coefficient as assessed by DW-MRI.

The primary aim of this study is to evaluate correlation between stage of hepatic fibrosis and liver apparent diffusion coefficient (ADC) and normalized liver ADC with spleen assessed by DW-MRI in patients with chronic viral hepatitis B or C.

***Secondary Outcome Measures:***

The optimal cutoff values of DW-MRI for determining the stage of liver fibrosis.

The aim of this study is to estimate the optimal cutoff values of DW-MRI for determining significant liver fibrosis (fibrosis stage ≥2) and advanced fibrosis (fibrosis stage ≥3).

The effects of hepatic steatosis, necroinflammation and hepatic iron on ADC values for determining the stage of liver fibrosis.

The aim of this study is to evaluate whether the degree of hepatic steatosis, necroinflammation, and hepatic iron affect the values of liver ADC and normalized liver ADC with spleen for determining the stage of liver fibrosis.

**Statistical analysis**

Descriptive statistics will be used to determine the characteristics of the patient population. Standard parametric and nonparametric statistics will be used for comparison of variables. The Spearman's rank correlation test will be used to assess the relationships of ADC values with the stage of liver fibrosis. The logistic regression model will be used to identify factors independently associated with discordance. The area under the receiver-operating characteristics (AUROC) curve will be constructed to evaluate the overall accuracy of DWI and will be compared between the ADCs at different *b*-values with the DeLong test. The optimal cutoffs of diffusion parameters for F2, F3, and F4 diseases will be chosen at points with the highest Youden’s index. Data analysis will be executed with the SPSS software package version 18.0 (SPSS Inc., Chicago, IL).

**References**

Taouli B, Tolia AJ, Losada M, Babb JS, Chan ES, Bannan MA, Tobias H. Diffusion-weighted MRI for quantification of liver fibrosis: preliminary experience. AJR Am J Roentgenol. 2007 Oct;189(4):799-806.

Liaw YF, Kao JH, Piratvisuth T, Chan HL, Chien RN, Liu CJ, Gane E, Locarnini S, Lim SG, Han KH, Amarapurkar D, Cooksley G, Jafri W, Mohamed R, Hou JL, Chuang WL, Lesmana LA, Sollano JD, Suh DJ, Omata M. Erratum to: Asian-Pacific consensus statement on the management of chronic hepatitis B: a 2012 update. Hepatol Int. 2012 Oct;6(4):809-10. doi: 10.1007/s12072-012-9386-z.

Soylu A, Kiliçkesmez O, Poturoğlu S, Dolapçioğlu C, Serez K, Sevindir I, Yaşar N, Akyildiz M, Kumbasar B. Utility of diffusion-weighted MRI for assessing liver fibrosis in patients with chronic active hepatitis. Diagn Interv Radiol. 2010 Sep;16(3):204-8. doi: 10.4261/1305-3825.DIR.2810-09.1. Epub 2010 Jul 25.

Ferraioli G, Tinelli C, Dal Bello B, Zicchetti M, Lissandrin R, Filice G, Filice C, Above E, Barbarini G, Brunetti E, Calderon W, Di Gregorio M, Gulminetti R, Lanzarini P, Ludovisi S, Maiocchi L, Malfitano A, Michelone G, Minoli L, Mondelli M, Novati S, Patruno SF, Perretti A, Poma G, Sacchi P, Zanaboni D, Zaramella M. Performance of liver stiffness measurements by transient elastography in chronic hepatitis. World J Gastroenterol. 2013 Jan 7;19(1):49-56. doi: 10.3748/wjg.v19.i1.49.

Talwalkar JA, Kurtz DM, Schoenleber SJ, West CP, Montori VM. Ultrasound-based transient elastography for the detection of hepatic fibrosis: systematic review and meta-analysis. Clin Gastroenterol Hepatol. 2007 Oct;5(10):1214-20. Review.

Wang Y, Ganger DR, Levitsky J, Sternick LA, McCarthy RJ, Chen ZE, Fasanati CW, Bolster B, Shah S, Zuehlsdorff S, Omary RA, Ehman RL, Miller FH. Assessment of chronic hepatitis and fibrosis: comparison of MR elastography and diffusion-weighted imaging. AJR Am J Roentgenol. 2011 Mar;196(3):553-61. doi: 10.2214/AJR.10.4580.

Bonekamp S, Torbenson MS, Kamel IR. Diffusion-weighted magnetic resonance imaging for the staging of liver fibrosis. J Clin Gastroenterol. 2011 Nov-Dec;45(10):885-92. doi: 10.1097/MCG.0b013e318223bd2c.

Tosun M, Inan N, Sarisoy HT, Akansel G, Gumustas S, Gürbüz Y, Demirci A. Diagnostic performance of conventional diffusion weighted imaging and diffusion tensor imaging for the liver fibrosis and inflammation. Eur J Radiol. 2013 Feb;82(2):203-7. doi: 10.1016/j.ejrad.2012.09.009. Epub 2012 Nov 2.

Onur MR, Poyraz AK, Bozdag PG, Onder S, Aygun C. Diffusion weighted MRI in chronic viral hepatitis: correlation between ADC values and histopathological scores. Insights Imaging. 2013 Jun;4(3):339-45. doi: 10.1007/s13244-013-0252-x. Epub 2013 May 11.

**Diffusion-weighted magnetic resonance imaging for assessing liver fibrosis in patients with chronic viral hepatitis**

**Demographics**

__ __ __ Patient’s code

__ __ Age (years)

__ Gender; 1 = Male, 2 = Female

**Clinical Details at the time of liver biopsy**

__ __.__ kg Body weight

__ __ __ cm Height

__ __ __ cm Neck circumference

__ __ __ cm Waist circumference

__ __ __ cm Hip circumference

__ Diabetes; 0= No, 1= IFG, 2= DM with diet controlled, 3= DM treated oral

hypoglycemic drugs, 4= Insulin requiring type 2 DM, 5= Type 1 DM

___ years Duration of diabetes before Liver biopsy

__ Dyslipidemia or Rx: 0= No, 1= Yes [chol __ __ __, tri __ __ __, HDL-C __ __ mg/dl]

__ Hypertensive: ≥130/85 or Rx, 0=No, 1=Yes [SBP __ __ __, DBP __ __ __ mmHg]

__ 0 = Nonsmoker, 1= Ex-smoker, 2= current smoker, if yes______pack years

**__** 0 = Nonalcohol, 1= Ex-drinking, 2= current drinking, if yes_______ gm/day

**Laboratory Data at time or closed to liver biopsy**

__ __.__ g/dL Hemoglobin

__ __ __ __/ml WBC

__ __ __ x 10^9^/L Platelet count

__ __ . __ sec Prothrombin time

__ . __ INR

__ __ __ g/dL Fasting blood sugar

__ __ __ mg/dL Cholesterol

__ __ __ mg/dL Triglyceride

__ __ __ mg/dL HDL-C

__ __ . __ mg/dL Total Bilirubin

__ __ __ U/L AST

__ __ __ U/L ALT

__ __ __ __ U/L Alkaline phosphatase

__ __ __ __ U/L GGT

__. __ g/dL Albumin

__. __ g/dL Globulin

__ __ __ __ mg/dL Serum ferritin

__ __ __ __ mg/dL Total iron binding capacity (TIBC)

__ __ __ __ mg/dL Serum iron

__ __ __ % Transferrin saturation

__ HBsAg; 0=negative, 1=positive

__ HBeAg; 0=negative, 1=positive

__ __ __ __ IU/ml HBV DNA viral load

__ Anti-HCV; 0=negative, 1=positive

__ __ __ __ IU/ml HCV RNA viral load

**Liver stiffness assessed by transient elastography**

__ __. __ kPa Liver stiffnes

__ __ __ IQ

__ __ __% Success rat

__ __ __ CAP value

**Liver stiffness assessed by shear wave elastography**

__ __. __ kPa Liver stiffness

**Radiological Data**

__ . __ __ __ __ mean liver ADC

__ . __ __ __ __ standard deviation of liver ADC

__ . __ __ __ __ mean spleen ADC

__ . __ __ __ __ standard deviation of spleen ADC

__ . __ __ __ __ ADC mean (liver/spleen)

**Histological interpretation**

__ __ / __ __ / __ __ __ __ Date of liver biopsy (MM/DD/YYYY)

__.__ cm Length of liver biopsy

__ __ Number of portal tract

__ Steatosis grade (0= <5%, 1= 5-33%, 2= >33-66%, 3= >66%)

__ Necroinflammatory grade (0= none, 1= mild, 2= moderate, 3= severe)

__ Fibrosis stage (0=none, 1= portal fibrosis without septa, 2= portal fibrosis with few septa, 3= numerous septa without cirrhosis, 4=cirrhosis)

**__** Total hepatic iron content score (0-33)

__ Hepatocyte iron score (0-18)

__ Sinusoid iron score (0-6)

__ Fibrosis iron score (0-9)
